# Molecular mechanisms underlying adverse effects of dexamethasone and betamethasone in the developing cardiovascular system

**DOI:** 10.1096/fj.202200676RR

**Published:** 2023-05-03

**Authors:** Tessa A. C. Garrud, Noor E. W. D. Teulings, Youguo Niu, Katie L. Skeffington, Christian Beck, Nozomi Itani, Fiona G. Conlon, Kimberley J. Botting, Lisa M. Nicholas, Wen Tong, Jan B. Derks, Susan E. Ozanne, Dino A. Giussani

**Affiliations:** ^1^ Department of Physiology, Development and Neuroscience University of Cambridge Cambridge UK; ^2^ Institute of Metabolic Science‐Metabolic Research Laboratories, MRC Metabolic Diseases Unit University of Cambridge, Addenbrooke's Hospital Cambridge UK; ^3^ Department of Perinatal Medicine University Medical Centre Utrecht Netherlands; ^4^ BHF Cardiovascular Centre for Research Excellence University of Cambridge Cambridge UK; ^5^ Strategic Research Initiative in Reproduction University of Cambridge Cambridge UK; ^6^ Centre for Trophoblast Research University of Cambridge Cambridge UK

## Abstract

Antenatal glucocorticoids accelerate fetal lung maturation and reduce mortality in preterm babies but can trigger adverse effects on the cardiovascular system. The mechanisms underlying off‐target effects of the synthetic glucocorticoids mostly used, Dexamethasone (Dex) and Betamethasone (Beta), are unknown. We investigated effects of Dex and Beta on cardiovascular structure and function, and underlying molecular mechanism using the chicken embryo, an established model system to isolate effects of therapy on the developing heart and vasculature, independent of effects on the mother or placenta. Fertilized eggs were treated with Dex (0.1 mg kg^−1^), Beta (0.1 mg kg^−1^), or water vehicle (Control) on embryonic day 14 (E14, term = 21 days). At E19, biometry, cardiovascular function, stereological, and molecular analyses were determined. Both glucocorticoids promoted growth restriction, with Beta being more severe. Beta compared with Dex induced greater cardiac diastolic dysfunction and also impaired systolic function. While Dex triggered cardiomyocyte hypertrophy, Beta promoted a decrease in cardiomyocyte number. Molecular changes of Dex on the developing heart included oxidative stress, activation of p38, and cleaved caspase 3. In contrast, impaired GR downregulation, activation of p53, p16, and MKK3 coupled with CDK2 transcriptional repression linked the effects of Beta on cardiomyocyte senescence. Beta but not Dex impaired NO‐dependent relaxation of peripheral resistance arteries. Beta diminished contractile responses to potassium and phenylephrine, but Dex enhanced peripheral constrictor reactivity to endothelin‐1. We conclude that Dex and Beta have direct differential detrimental effects on the developing cardiovascular system.

## Graphical abstract

Antenatal glucocorticoids trigger adverse effects on the developing cardiovascular system, but mechanisms are unknown. We investigated effects of Dexamethasone (Dex) and Betamethasone (Beta) using the chicken embryo, an established model to isolate effects of therapy, independent of the mother and placenta. While Dex triggered cardiomyocyte hypertrophy, Beta reduced cardiomyocyte number. Dex activated oxidative stress, p38 and caspase 3. In contrast, impaired GR downregulation, p53 activation and CDK2 repression linked the effects of Beta on cardiomyocyte senescence and greater cardiac dysfunction.

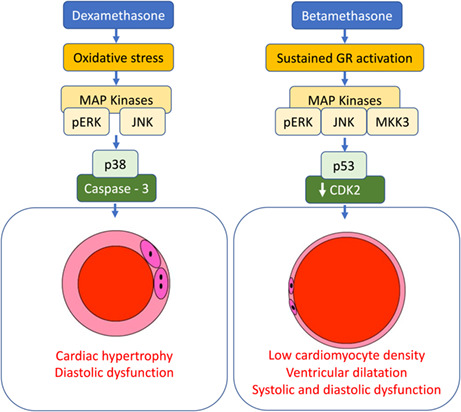

**Abbreviations**
2^−ΔΔCT^
: method of presenting changes in level of gene expression by qRT‐PCR
a/p: the area that each point represents
ACh: acetylcholine
AGT: antenatal glucocorticoid therapy
AWERB: Animal Welfare and Ethical Review Body
Beta: betamethasone
BSA: bovine serum albumin
ca.: circa
CaCl_2_·2H_2_O: calcium chloride dihydrate
CAST: computer‐assisted stereological toolbox
cdk2: cyclin‐dependent kinase 2
Dex: dexamethasone
ERK: extracellular signal‐regulated kinase
ET‐1: Endothelin 1
GR: glucocorticoid receptor
H: the z‐height of the dissector
HEPES: a zwitterionic organic chemical buffering agent
HPRT: genomic housekeeper gene
HR: heart rate
HSP27: heat shock protein 27
HSP60: heat shock protein 60
HSP70: heat shock protein 70
IgG: immunoglobulin G
JNK: jun amino‐terminal kinase
KCl: potassium chloride
KH_2_PO_4_
: potassium dihydrogen sulfate
kPa: kilopascal
LVDP: left ventricular developed pressure
LVEDP: left ventricular end diastolic pressure
LVSP: left ventricular systolic pressure
M: molar
MgCl_2_
: magnesium chloride
MgSO_2_
: magnesium sulfate
miRNeasy: kit used to purify total RNA
MKK3: mitogen‐activated protein kinase kinase 3
mRNA: messenger RNA
NaCl: sodium chloride
NaHCO3: sodium bicarbonate
NO: nitric oxide
NIH: National Institutes of Health
p16: a tumor suppressor/marker of cell senescence
p38: a class of mitogen‐activated protein kinase
p53: a tumor suppressor/marker of cell senescence
PDI: protein disulfide isomerase
PE: phenylephrine
pERK: phosphorylated extracellular signal‐regulated kinase
PFA: paraformaldehyde
pJNK: phosphorylated jun amino‐terminal kinase
pSAPK: phosphorylated stress‐activated protein kinase
Q: total number of nuclei counted
qRT‐PCR: quantitative reverse transcription‐polymerase chain reaction
RNA: ribonucleic acid
SAPK: stress‐activated protein kinase
Sdha: succinate dehydrogenase complex flavoprotein subunit A
SEM: standard error of the mean
SNP: sodium nitroprusside
SP‐B: surfactant protein B
TBS: tris‐buffered saline
TBS‐T: tris‐buffered saline and polysorbate
w/v: weight for volume
β_1_
: beta 1
ΣP: the sum of points that lie over tissue

## 1 INTRODUCTION

Antenatal glucocorticoid therapy (AGT) has been used extensively and successfully in the clinic for the treatment of threatened preterm birth since the 1990s.^1^ AGT substantially reduces the risk of mortality and severe morbidity in the preterm infant, such as respiratory distress syndrome (RDS) due to lung immaturity.^2^ However, AGT can also trigger adverse effects on other organs, such as the developing cardiovascular system.^3^, ^4^ This has raised interest in how to optimize current AGT regimens to maintain benefits while minimizing off‐target side effects.^5^, ^6^ Notably, two different synthetic steroids dexamethasone (Dex) and betamethasone (Beta) are used globally and interchangeably, with no consensus on the optimal drug of choice.^7^ Both are analogs of cortisol with fluorination at the 9‐C position and insertion of the 1,2 carbon–carbon double bond. Due to these additions, Dex and Beta have negligible mineralocorticoid activity, but their glucocorticoid potency is approximately 25‐fold higher than that of natural cortisol.^8^, ^9^

Clinical studies have compared the efficacy of Dex versus Beta when treating preterm birth. Some show that Beta can better prevent RDS and neonatal death, while others support that Dex is superior at preventing intraventricular hemorrhage.^10^, ^11^, ^12^ There are also studies that have reported no observable differences between the two drugs.^13^ More recently, the ASTEROID study designed to directly compare Dex and Beta for use in AGT, reported no difference in death or neurosensory disability in children at age 2 years between the two groups.^14^ However, a recent systematic review suggested possible clinically relevant differences in outcomes for chorioamnionitis, fetal death, and RDS following Dex or Beta administration.^15^ Experimental studies in sheep and primates have also shown differing effects of Dex versus Beta in terms of pulmonary outcomes.^16^, ^17^, ^18^ Combined, therefore, the relatively small numbers of clinical and experimental studies comparing Dex and Beta make it difficult to assess what the best treatment, in relation to short‐term outcomes, for AGT should be. Indeed, both the UN committee on maternal and neonatal health, and a report by the WHO on preventing preterm birth, conclude that there is inadequate evidence to recommend one over the other, and that more research on underlying mechanisms is sorely needed.^19^, ^20^ One mechanism leading to glucocorticoid‐induced cardiovascular dysfunction is the generation of oxidative stress.^21^, ^22^, ^23^, ^24^, ^25^ Glucocorticoids are potent inducers of reactive oxygen species (ROS), and they can trigger hypertension and endothelial dysfunction, effects that can all be prevented by antioxidant treatment.^23^, ^24^, ^26^, ^27^, ^28^, ^29^, ^30^ Therefore, it is possible that mechanisms mediating off‐target adverse effects of synthetic glucocorticoids relate to the induction of excess ROS production and associated intracellular signaling, including the activation of stress kinases, cell cycle changes, and the induction of cell senescence.^25^, ^31^

Since AGT in human practice is administered to the mother by intramuscular injection, possible differential effects of Dex versus Beta may relate to alterations in the physiology of the mother, placenta, and/or offspring. For instance, maternal but not fetal treatment with synthetic glucocorticoids reduces fetal growth.^32^ In addition, maternal treatment with Dex increases uteroplacental vascular resistance.^33^ Both these studies support effects of AGT at the level of the placenta, impairing placental perfusion.

Therefore, the aim of this study was to isolate effects on the developing cardiovascular system of Dex compared with Beta and to determine underlying molecular changes to cellular proliferation, senescence, and stress pathways, independent of effects on the mother or placenta using the chicken embryo model system. This directly addresses the knowledge deficit identified by the WHO in relation to AGT. Treatment started at day 14/21–day incubation period. This time window is comparable to ca. 27 weeks of human pregnancy, a stage in gestation when AGT is administered when preterm birth is threatened. Outcomes were studied immediately before term on day 19. We adopted an integrative approach, combining experiments at the isolated organ, cellular, and subcellular levels to test the hypothesis that treatment of the chicken embryo with either glucocorticoid will have adverse effects on the developing cardiovascular system, however, the specific effects on cellular proliferation, senescence, and stress pathways will differ between the drugs.

## 2 METHODS

### 2.1 Ethics statement

All research was approved under the Animals (Scientific Procedures) Act 1986 (Amendment Regulations 2012) by the University of Cambridge Animal Welfare and Ethical Review Body (AWERB).

### 2.2 Experimental procedure

Fertilized Bovans Brown eggs (Medeggs, Henry Stewart & Co., UK) were delivered weekly and stored at 14°C to arrest development, as commonly practiced.^34^, ^35^, ^36^, ^37^, ^38^ Eggs were accumulated over 3–5 days at this temperature to synchronize development, prior to incubation. The supplier delivered the fertilized eggs in batches from a single day's laying, meaning that each egg used in the present program of work came from a different hen. Eggs were placed in an incubator under normoxic (21% O_2_) optimal conditions (37.9°C, 45% humidity, 12:12 h light: dark cycle, automatic rotation every hour, and incubator Mod.75‐A and Mod.M‐240, Masalles, Spain). This was termed as day 1 and the start of the incubation period. A dose of 0.1 mg/kg in 0.1 mL of water vehicle of either dexamethasone (Na^+^‐phosphate form, Sigma Aldrich) or betamethasone (Celestone soluspan, Na^+^phosphate and acetate, Merck) was injected on day 14 out of the 21‐day total incubation period. The control group received 0.1 mL of water. To dose the eggs, drug administration was made through a small hole in the shell topically onto the chorioallantoic membrane with substances being absorbed by the chorioallantoic circulation. Our previous studies have established this as an effective noninvasive method of egg dosing.^25^, ^36^, ^37^, ^38^ To determine the amount of Dex or Beta needed, a pilot study was conducted where the weight of 5 × day 14 embryos was determined. The mean weight of these five embryos was then used to calculate the dose of 0.1 mg/kg of glucocorticoid needed. The dose of 0.1 mg/kg used was based on our previous work in fetal sheep^39^, ^40^ and available human clinical information. Pregnant women selected for AGT receive 24 mg of either Dex or Beta over a 48 h period.^41^ It is known that approximately one third of maternal treatment with synthetic glucocorticoids crosses the human placenta.^42^ Assuming an average pregnant woman weighs 70 kg, this yields 0.1 mg/kg of synthetic glucocorticoid that will reach the human fetus. Four cohorts of chicken embryos were used to achieve significant power per outcome per group. Cohort 1 was used for biometry and snap‐freezing tissues for molecular studies. Cohort 2 was used for the isolated Langendorff preparation and small vessel wire myography. Cohort 3 was used for perfusion fixation. Cohort 4 was used for molecular digestion of the embryonic heart to isolate individual cardiomyocytes.

### 2.3 Biometry

On day 19 of incubation, egg mass was measured in the chicken embryo (Controls, *n* = 10; Dex, *n* = 10; and Beta, *n* = 15). Embryos were then removed from the egg and killed by spinal cord transection. The mass of the embryo, the brain, and the heart was noted.

### 2.4 Langendorff preparation

On day 19, embryos (Controls, *n* = 10; Dex, *n* = 10; and Beta, *n* = 9) were removed from their eggshells and killed by spinal cord transection. The hearts were then mounted on the Langendorff apparatus, as described previously.^25^, ^36^, ^37^, ^38^ Baseline readings of several variables were taken (left ventricular end diastolic pressure, LVEDP; left ventricular developed pressure, LVDP; Cycle duration; diastolic and systolic cycle duration; Tau, τ; and the Contractility Index;). In vitro responses to the sympathetic β_1_‐adrenergic receptor agonist isoprenaline (Sigma, UK, in the range 10^−9^–10^−7^ M) and the parasympathetic cholinergic agonist Carbachol (Sigma, UK, in the range 10^−8^–10^−6^ M) were then determined by administering doses in random order to the heart via the perfusing cannula. The heart was given time to return to baseline measurements between each dose.

### 2.5 Cardiac stereology

At day 19, another group of embryos (Controls, *n* = 8; Dex, *n* = 8, Beta, *n* = 10) was placed under terminal anesthesia (0.3 mL intraperitoneal Pentobarbitone Sodium 20%, National Veterinary Services Limited). The chest cavity was exposed, and a cardiac puncture made into the left ventricle. The circulatory system was perfused with heparinized saline solution, then with 10% PFA for approximately 30 min at a physiological pressure of 2.66 kPa.^25^, ^37^, ^38^ The heart and ascending aorta were dissected, placed into 10% PFA for 24 h, and then transferred to PBS and stored at 4°C. Fixed hearts were sectioned and slices arranged in ascending order (apex side down) and photographed. Images were analyzed using the ImageJ software (version 1.46, National Institute of Health, USA). To measure areas, a cross grid of 1 mm^2^ was superimposed onto the images and crosses within each compartment (left ventricle: LV; left lumen: LL; right ventricle: RV; and right lumen: RL) were scored if the center of the cross was on the compartment. Compartment areas were assessed in all heart sections. Areas were then calculated according to the Cavalieri principle,^43^ using:

$$\text{Area}\;\left({\mathrm{mm}}^{2}\right)=A\left(\rho \right)*\sum P$$

$$\text{Volume}\;\left({\mathrm{mm}}^{3}\right)=\text{area}*\text{section thickness}*\mathrm{no}.\;\text{of sections}$$

Where A(ρ) is the area associated with each point, and ΣP is the number of points in each compartment.

The initial 5 mm of the ascending aorta was dissected from the top of each fixed heart and then embedded into a paraffin block and sectioned at 5 μm. Consecutive sections (*n* = 10) for each aorta segment were arranged onto a microscope slide and then stained with Gill's Hematoxylin (Sigma, UK). Gridlines of 100 μm were imposed on the images and measurements were made at gridline intersections with the outside of the blood vessels. To quantify area, a grid of points was placed over the image and values calculated using the Cavalieri principle, as described above.

Following stereological assessment, the left ventricle was cut into 1 mm^3^ cubes, embedded in glycolmethacrylate resin, and then sectioned at 30 μm thickness. To identify cardiomyocyte nuclei, the sections were stained with hematoxylin and 0.15% Basic Fuchsine (Sigma‐Aldrich, Gillingham, UK). The numerical density of cardiomyocyte nuclei was established using the optical dissector technique.^25^, ^37^, ^38^ NewCast software (Computer Assisted Stereological Toolbox, Visopharm Integrator system software version 4.6.3.857) randomly, systematically, and uniformly assigned a counting frame to each ventricle piece. Point counting was performed using 15 frames, each covering an area of 400 μm^2^, and a dissector height of 10 μm. To determine the numerical density of cardiomyocyte nuclei, the following equation was used:

$$\text{Cardiomyocyte number}\;\mathrm{per}\;\mu m^{3}\text{left ventricle}=\frac{\Sigma Q}{h\times \left(\mathrm{ap}\right)\mathrm{\Sigma P}\;}$$

Where *Q* is the total number of nuclei counted, *h* is the *z*‐height of the dissector, ap is the area of ventricle that each point represents, and ΣP is the sum of points that lie over ventricular tissue.

### 2.6 Cardiomyocyte cell size

In another cohort (Controls: *n* = 7; Dex: *n* = 6, and Beta: *n* = 5), individual cardiomyocytes were isolated on day 19 via enzymatic degradation, as described previously.^25^ The coronary vasculature was perfused via the aorta with Tyrode's solution (140 mM NaCl, 5 mM KCl, 1 mM MgCl_2_, 10 mM Glucose, 10 mM HEPES, and pH 7.35) for several minutes until blanched. Type II collagenase (160 U/mL, Worthington) and protease type XIV from *Strep. griseus* (0.78 U/mL, Sigma), were then added to the perfusing solution for around 10 min. The heart was then perfused with Kreb's buffer (50 mM L‐Glutamic Acid, 30 mM KCl, 20 mM Taurine, 0.5 mM EGTA, 10 mM HEPES, 10 mM Glucose, 30 mM KH2PO4, 3 mM MgSO4, and pH 7.37) for a further 10 min. Hearts were then placed into roughly 5 mL of Kreb's buffer and shaken gently to release cells, then left to settle for 45 min. PFA (10% Sigma) was added to reach a 4% concentration, which was then stored at room temperature. Solutions were shaken, then 1 mL of cell suspension was mixed with 1 mL of Methyl‐Blue stain (Sigma), and visualized under a light microscope. The Computer Assisted Stereological Toolbox (NewCast software, Visiopharm Integrator system version 4.6.3.857) was used to randomly search each solution sample to measure 50 randomly chosen cardiomyocytes per heart. Cardiomyocytes were then assessed for nuclearity, width, and length, and cardiomyocyte volume was estimated using the formula volume = 2/3π*lw2/4, where l is length, and w is width, as before.^25^

### 2.7 Quantification of mRNA expression, protein carbonylation, and western blot

At day 19 following killing of the embryo (Controls: *n* = 10; Dex: *n* = 7 and Beta: *n* = 7), hearts were excised and snap frozen. Frozen whole hearts were then powdered and aliquoted into 50 mg samples for RNA and protein extraction. Cardiac RNA was extracted using the miRNeasy Mini Kit (Qiagen, Hilden, Germany). Complementary DNA (cDNA) was generated from 3 μg RNA per sample using Thermo Scientific RevertAid First Strand cDNA Synthesis kit (Thermo Scientific, Waltham, Massachusetts, USA). Real‐time quantitative PCR was performed using SYBR Green master mix and specific primers. Gene expression was normalized to the geometric mean of housekeepers *HPRT* and *Sdha*, expression of which were no different between groups. The relative fold change (2^−ΔΔCT^) was then assessed. The forward and reverse primer sequences are shown in Supporting Information Table S1. To determine the extent of posttranslational protein carbonylation that occurs as a result of oxidative damage to proteins, an OxyBlotTM analysis was performed using an OxyBlot detection kit, according to the manufacturer's specifications (Millipore, Billerica, MA).^25^ Remaining snap‐frozen tissue was used to extract proteins using cell lysis buffer (10× solution, Cell Signalling, UK) with addition of a protease inhibitor cocktail (cOmplete Mini, EDTA‐free, Roche Diagnostics, Dl). Western blotting was then performed as described previously.^36^, ^37^, ^38^ Briefly, protein samples in SDS buffer were boiled at 90°C for 10 min and then allowed to cool. Samples were then loaded into the gel wells at a total protein amount of 15 μg per well. Gels were run at 110 V, 12 mA for 4 h. Samples were then transferred from the agarose gel to a membrane (Polyvinyl difluoride Immobilon‐P membrane, Millipore, USA), using a semidry transfer apparatus (Bio‐Rad semi‐dry transfer pack, Bio‐Rad Laboratories, UK). Once this was completed, the membrane was removed, stained with Ponceau S solution (Sigma, UK) for 5 min, and an image was taken. Subsequently, the membrane was washed in TBST, then soaked for 1 h in blocking buffer at room temperature prior to antibody staining. Primary antibodies (HSP27 Cell Signaling Technology 2402S, HSP60 Abcam ab46798, HSP70 Cell Signaling Technology 4872, phosphorylated ERK1/2 Cell Signaling Technology 9101S, ERK1/2 Cell Signaling Technology 9102, SAPK/JNK Cell Signaling Technology 2402S, phosphorylated SAPK/JNK Cell Signaling Technology 99251S, PDI Abcam ab3672, GR Santa Cruz Biotechnology sc‐393 232, and cleaved caspase‐3 BD Biosciences 610 539) were diluted in either 5% BSA, or 5% milk powder (Marvel Original, Premier Foods, UK), and the membrane was incubated overnight at 4°C. Secondary antibody incubation was performed at room temperature for 1 h. TBST was used to wash the membranes between blocking, primary and the secondary incubation steps. Antibodies were detected by using West Pico chemiluminescent substrate (Thermo Scientific, UK), then exposing the membranes to a photosensitive film (GE Healthcare Amersham Hyperfilm ECL, Fisher Scientific, UK). The intensity of the bands was assessed using ImageJ software (version 1.46, National Institute of Health, USA), and normalized to the total protein expression determined by the Ponceau stain.

### 2.8 Assessment of vascular reactivity

In another cohort of embryos (Controls: *n* = 10; Dex: *n* = 10, and Beta: *n* = 10), a second‐order branch of the femoral artery (diameter ca. 300 μm) was dissected and placed in cold Krebs buffer (118.5 mM NaCl, 25 mM NaHCO3, 4.7 mM KCl, 1.2 mM MgSO4.7H2O, 1.2 mM KH2PO4, 2.5 mM CaCl2, and 2.8 mM D‐glucose; all reagents Sigma, UK). Vasomotor reactivity was then assessed using a wire myograph apparatus, as described previously (620 M, DMT).^23^, ^37^, ^38^ To determine vasodilator function, vessels were pre‐constricted with 64 mM KCl to induce a submaximal constriction, then exposed to doses of acetylcholine (ACh, 10^−9^–10^−4^ M) to determine endothelium‐dependent relaxation, or sodium nitroprusside (SNP, 10^−9^–10^−4^ M) to determine vascular smooth muscle‐dependent relaxation. To determine the partial contributions of endogenous NO‐dependent and NO‐independent mechanisms to the ACh‐induced vasorelaxation, the ACh dose–response curve was repeated after incubating the vessel with L‐NAME (10^−5^ M, 10 min). Then, using established methodology,^23^, ^44^ the contribution of NO to the vascular relaxation induced by ACh was calculated by subtracting the area above the curve (AAC) for ACh—the AAC for ACh + LNAME. The remaining AAC represented the contribution of NO‐independent mechanisms to the vascular relaxation induced by ACh. Vascular constrictor reactivity was assessed with increasing doses of K^+^ solutions (16.74–250 mM), of phenylephrine (PE, 10^−8^–10^−4^ M) and of endothelin (ET, 10^−9^–10^−6^ M). The response to K^+^ was normalized to the diameter of the vessel (mN mm 1 μm 1.1000 1). The response to PE and ET were normalized to the response to 125 mM K^+^ of the same vessel (%K^+125^). LabChart was used for data acquisition and analysis (Labchart 6.0, Powerlab 8/30; AD Instruments, Chalgrove, UK).

### 2.9 Assessment of lung maturation

At day 19, following perfusion fixation, the embryonic lungs were dissected in another cohort of animals (Controls: *n* = 8; Dex: *n* = 8, and Beta: *n* = 6). The lungs were placed in 4% PFA, then changed to PBS after 24 h. Samples were embedded in paraffin and then sectioned at a thickness of 8 μm. Sectioning began at the posterior end of the embedded lungs, 10 tissue sections were taken, with the seventh and eighth chosen for analysis. Slides were stained with the same protocol used for cardiomyocyte nuclear density analysis. For each lung section, a total of 20 measurements were made for the air capillary diameter, and the distance between the air capillary to the nearest blood vessel, equating to the diffusion distance. At day 19, another cohort of embryos (Controls, *n* = 6; Dex, *n* = 6 Beta, *n* = 6) was killed via spinal cord transection and both left and right lung lobes were dissected, weighed, and snap frozen in liquid nitrogen. Roughly, 50 mg of lung tissue was homogenized and assayed for protein concentration. A total quantity of 15 mg protein per sample was run on a western blot, and then transferred to a nitrocellulose membrane. Total protein loading was assayed with Ponceau S (Sigma, UK) staining of the membrane. Membranes were incubated overnight in primary antibody (SP‐B Stratech 365 148, Caveloin‐1 Thermo Fisher PA5‐17447), and then incubated with a HRP‐conjugated secondary antibody. Antibodies were detected using West Pico chemiluminescent substrate (Thermo Scientific). Band intensity was assessed using ImageJ software (NIH) and normalized to total protein.

### 2.10 Plasma glucocorticoid concentrations

In a final group of embryos (Controls: *n* = 8; Dex: *n* = 8, and Beta: *n* = 8), chorioallantoic fetal blood was taken 24 h after treatment for measurement of Dex or Beta in the circulation. Plasma concentrations of the two glucocorticoids were determined by liquid chromatography–tandem mass spectrometry (LC/MS/MS). The injection volume was 10 μL, the running time was 6.5 min for each injection, and the samples were kept in dark autosampler vials <4°C before determination. Differential retention time of samples ensured specificity and the absence of the interference of metabolites and decomposition products. The lower limit of quantification was 0.2–0.3 ng.ml^−1^ for both glucocorticoids.

### 2.11 Data and statistical analysis

Appropriate power calculations derived from previous datasets were performed to determine the minimum sample size required to achieve statistical significance set at *p* < .05.^25^, ^36^, ^37^, ^38^ Eggs were chosen for treatment using a random number generator. Wherever possible, scientists measuring outcomes were blinded to treatments. Data were analyzed using GraphPad Prism v5.0. Data are presented as mean ± SEM unless otherwise stated. Groups were statistically compared via one‐way ANOVA with Tukey post hoc test, or mixed model two‐way ANOVA, where appropriate. For all comparisons, statistical significance was accepted when *p* < .05.

## 3 RESULTS

### 3.1 Plasma glucocorticoid concentrations

Dex or Beta were undetectable in plasma from control embryos. The plasma Dex concentration achieved 24 h after administration in the Dex‐treated embryos was 2.8 ± 0.1 ng mL^−1^ (*n* = 8; mean ± SEM). The plasma Beta concentration achieved 24 h after administration in the Beta‐treated embryos was 2.1 ± 0.1 ng mL^−1^ (*n* = 8; mean ± SEM).

### 3.2 Biometry

**FIGURE 1:**
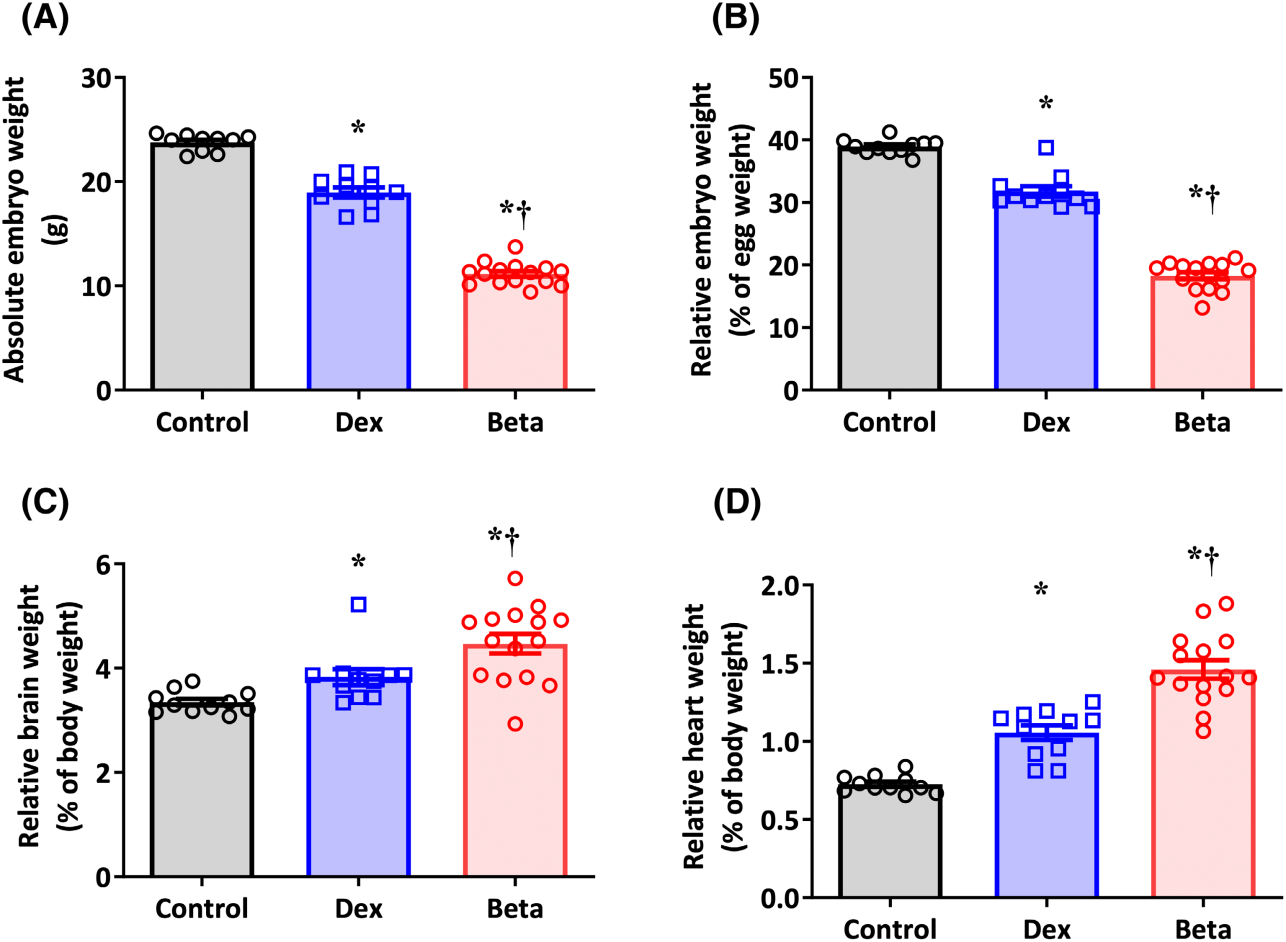
Effect of Dex and Beta on biometry in the chicken embryo. Data are mean ± SEM for chicken embryos at day 19 of incubation following vehicle (Control: *n* = 10), dexamethasone (Dex: *n* = 10), or betamethasone (Beta: *n* = 15) treatment at day 14 of incubation. (A) Absolute embryo weight in grams; (B) Embryo weight relative to initial egg weight; (C) Absolute heart weight in grams; (D) Heart weight relative to body weight. Significant differences are (*p* < .05): *versus Control; †Beta versus Dex. One‐way ANOVA with Tukey post hoc test.

Compared with controls, chicken embryos treated with Dex or Beta had significantly reduced body weight on day 19 of incubation, both in absolute terms as well as relative to initial egg mass (Figure 1A,B). Compared with controls, chicken embryos treated with Dex or Beta showed a significant increase in brain and heart mass, relative to total body mass (Figures 1C,D). For all biometry comparisons, the effects of Beta were significantly greater than Dex (Figure 1A–D).

### 3.3 Cardiac function

**FIGURE 2:**
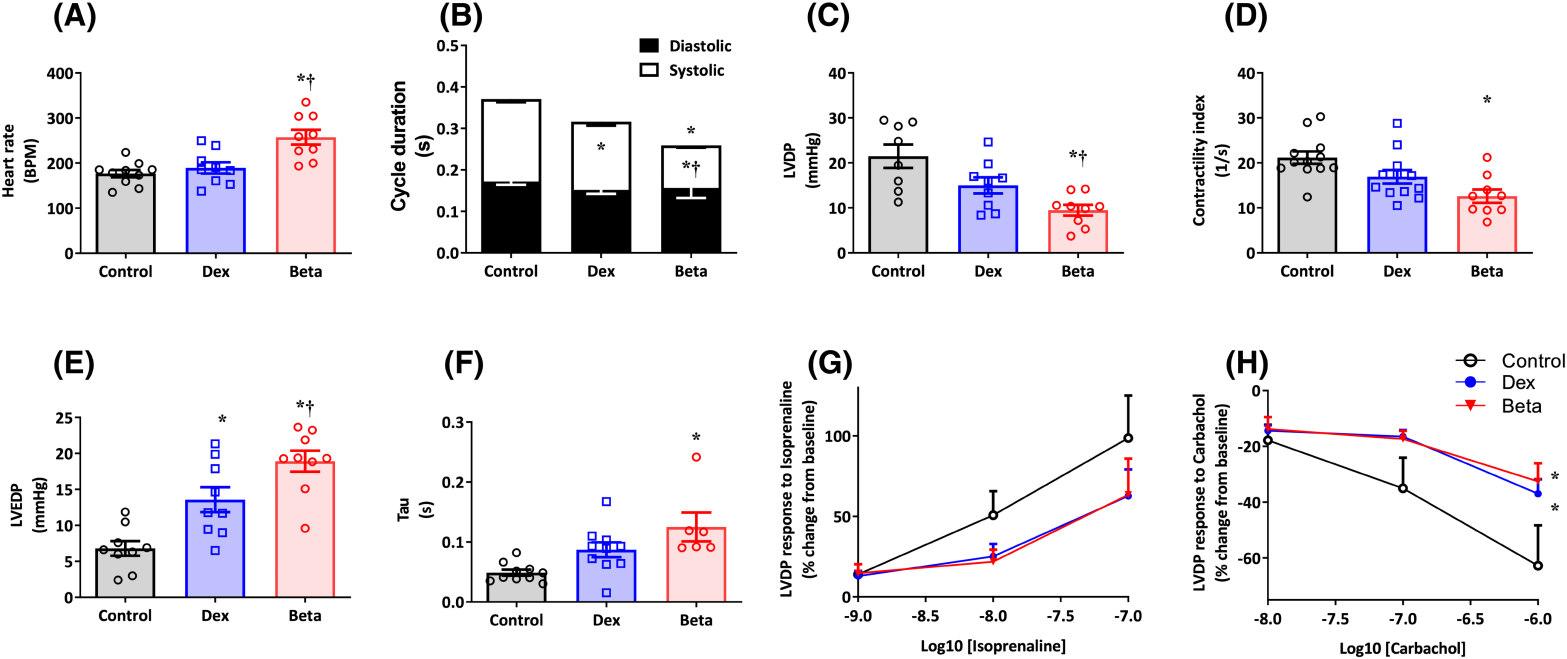
Effect of Dex and Beta on cardiac function in the chicken embryo. Data are mean ± SEM for chicken embryos at day 19 of incubation following vehicle (Control: *n* = 10), dexamethasone (Dex: *n* = 10), or betamethasone (Beta: *n* = 9) treatment at day 14 of incubation. (A) Heart rate; (B) Cardiac cycle duration (overall histogram describes overall cycle duration, black histogram is the diastolic component, and white histogram is the systolic component); (C) Left ventricular developed pressure (LVDP); (D) Contractility index; (E) Left ventricular end diastolic pressure (LVEDP); (F) Left ventricular relaxation time constant (Tau); (G,H) Left ventricular inotropic responses to isoprenaline and carbachol (Control: open symbols; Dex: blue; and Beta: red). Significant differences are (*p* < .05): *versus Control; ^†^Beta versus Dex. One‐way ANOVA with Tukey post hoc test. One‐way ANOVA with Tukey post hoc test for Panels A–F; Two‐way RM ANOVA with Tukey post hoc test for Panels G and H.

The Langendorff preparation revealed that hearts from embryos treated with Beta had a significantly greater basal heart rate compared to control embryos or those treated with Dex, corresponding with a significantly reduced systolic component of the cardiac cycle duration (Figure 2A,B). Compared with controls, chicken embryos treated with Beta but not Dex had significantly lower indices of systolic function, such as a fall in left ventricular developed pressure (LVDP) and in the contractility index (Figure 2C,D). Compared with controls, chicken embryos treated with either Dex or Beta had significantly enhanced left ventricular end diastolic pressure (LVEDP), an index of diastolic impairment. However, the effect on LVEDP was greater in embryos treated with Beta compared with those treated with Dex (Figure 2E). Compared with controls, only embryos treated with Beta but not Dex showed an elevation in Tau, another index of diastolic dysfunction (Figure 2F). Compared with controls, chicken embryos treated with either Dex or Beta had significantly diminished LVDP responses to the parasympathetic muscarinic receptor agonist carbachol. Any change in LVDP reactivity to the sympathetic beta‐receptor agonist isoprenaline did not reach significance for either treatment group (Figure 2G,H).

### 3.4 Cardiac stereology, aortic histology, and cardiomyocyte analysis

**FIGURE 3:**
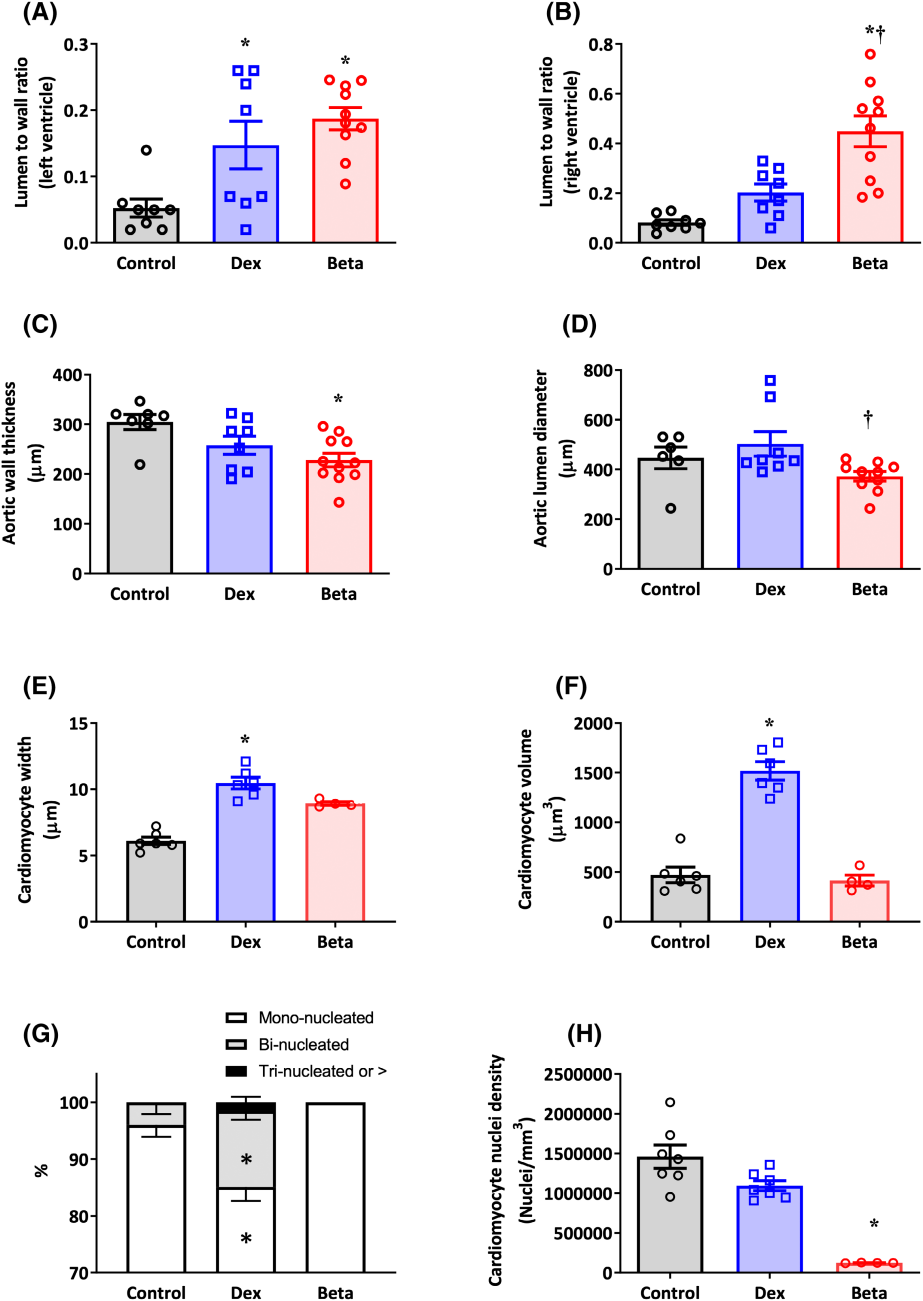
Effect of Dex and Beta on cardiovascular morphology in the chicken embryo. Data are mean ± SEM for chicken embryos at day 19 of incubation following vehicle (Control: *n* = 7–8), dexamethasone (Dex: *n* = 6–8), or betamethasone (Beta: *n* = 5–10) treatment at day 14 of incubation. (A) Ratio of the lumen to wall volume in the left ventricle; (B) Ratio of the lumen to wall volume in the right ventricle; (C) Aortic wall thickness in micrometers; (D) Aortic lumen diameter in micrometers; (E) Width of cardiomyocytes in micrometers; (F) Cardiomyocyte volume in cubic micrometers; (G) Percentage of mononucleated cardiomyocytes; (H) Cardiomyocyte nuclei density per cubic millimeter. Significant differences are (*p* < .05): *versus Control; ^†^Beta versus Dex. One‐way ANOVA with Tukey post hoc test.

Compared with controls, chicken embryos treated with either Dex or Beta had a significantly greater lumen to wall ratio in the left ventricle (Figure 3A). Compared with controls, chicken embryos treated with Beta but not Dex showed a much greater lumen to wall ratio in the right ventricle (Figure 3B). Compared with controls, chicken embryos treated with Beta but not Dex showed a significant reduction in aortic wall thickness (Figure 3C). Aortic lumen diameter was significantly smaller in chicken embryos treated with Beta compared with those treated with Dex (Figure 3D). Compared with controls, chicken embryos treated with Dex but not Beta showed an increase in cardiomyocyte width and volume (Figure 3E,F). Compared with controls, mononucleation was significantly decreased in chicken embryos treated with Dex but not Beta (Figure 3G). Conversely, compared with controls, cardiomyocyte nuclear density was significantly decreased in chicken embryos treated with Beta but not Dex (Figure 3H).

### 3.5 Cardiac markers of oxidative stress and activation of cell senescence signaling

**FIGURE 4:**
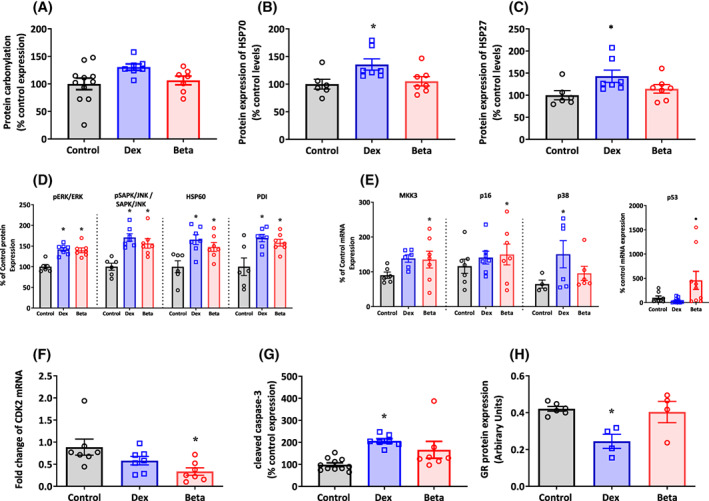
Effect of Dex and Beta on cardiac molecular pathways in the chicken embryo. Data are mean ± SEM for chicken embryos at day 19 of incubation following vehicle (Control: *n* = 6–11), dexamethasone (Dex: *n* = 7), or betamethasone (Beta: *n* = 7) treatment at day 14 of incubation. (A) Protein carbonylation; (B) Protein expression of HSP70; (C) Protein expression of HSP27; (D) Protein expression of the ratio of pERK to ERK, the ratio of pSAPK/JNK to SAPK/JNK, the protein expression of HSP60, and the protein expression of PDI; (E) mRNA expression of MKK3, p16, p38, and p53; (F) mRNA expression of CDK2; (G) Protein expression of cleaved Caspase‐3; (H) GR protein levels relative to tubulin. Significant differences are (*p* < .05): *versus Control; One‐way ANOVA with Tukey post hoc test.

Compared with controls, chicken embryos treated with Dex but not Beta showed an increase in the protein expression of HSP70 and HSP27, with no differences in cardiac protein carbonylation levels between groups (Figure 4A–C). Compared with controls, chicken embryos treated with either Dex or Beta showed an increase in the pERK/ERK protein ratio, and in the pSAPK/JNK to SAPK/JNK ratio (Figure 4D). Compared with controls, chicken embryos treated with either Dex or Beta also showed an increase in the cellular expression of the chaperone proteins HSP60 and PDI (Figure 4D). Compared with controls, chicken embryos treated with Beta but not Dex showed a significant increase in the mRNA expression of MKK3, p16, p53, and a fall in the mRNA expression of CDK2 (Figure 4E,F). Conversely, compared with controls, chicken embryos treated with Dex but not Beta showed an increase in the mRNA expression of p38 and cleaved caspase‐3 (Figure 4E,G), and a fall in the cardiac expression of the GR protein level (Figure 4H). There are a couple of instances (e.g., Panel 4E for MKK3 and P16, and Panel 4F) in which there may be a difference between control chicken embryos and those treated with Dex, but due to the effect size being small, we were not able to detect a significant difference in this study. Further, the protein/RNA analyses did not distinguish between left ventricle or right ventricle.

### 3.6 Vascular function

**FIGURE 5:**
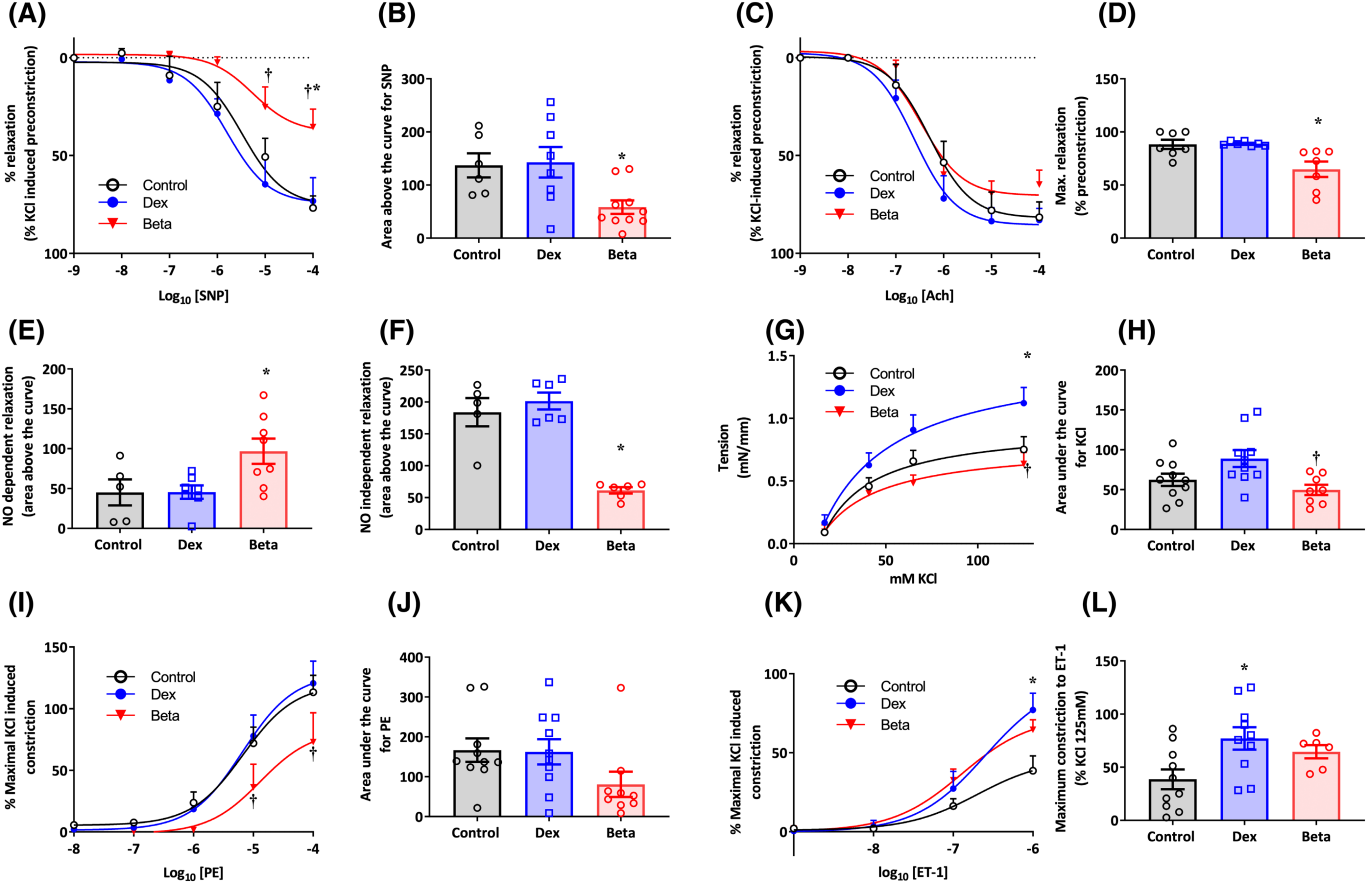
Effect of Dex and Beta on peripheral vascular reactivity in the chicken embryo. Data are mean ± SEM for chicken embryos at day 19 of incubation following vehicle (Control: *n* = 5–10), dexamethasone (Dex: *n* = 6–10), or betamethasone (Beta: *n* = 6–10) treatment at day 14 of incubation. (A) Vasorelaxant response to SNP (Control: open symbols; Dex: blue; and Beta: red); (B) Area above the curve for vasodilatation in response to SNP; (C) Vasorelaxant response to ACh (Control: open symbols; Dex: blue; and Beta: red); (D) Maximal vasodilatation in response to ACh; (E) Area for the NO‐dependent relaxation in response to ACh following treatment of the vessels with the NOS inhibitor L‐NAME; (F) Area for the NO‐independent relaxation in response to ACh following treatment of the vessels with the NOS inhibitor L‐NAME; (G) Constrictor response curves in response to K^+^ (Control: open symbols; Dex: blue; and Beta: red); (H) Area under the curve for vasoconstriction in response to K^+^; (I) Constrictor response curves in response to PE (Control: open symbols; Dex: blue; and Beta: red); (J) Area under the curve for vasoconstriction in response to PE; (K) Constrictor response curves in response to ET‐1 (Control: open symbols; Dex: blue; and Beta: red); (L) Maximal vasoconstriction in response to ET‐1. Significant differences are (*p* < .05): *versus Control; ^†^Beta versus Dex. One‐way ANOVA with Tukey post hoc test. One‐way ANOVA with Tukey post hoc test for Panels B, D, E, F, G, J, and L; Two‐way RM ANOVA with Tukey post hoc test for Panels A, C, G, I and K.

Compared with controls, femoral vessel segments isolated from chicken embryos treated with Beta but not Dex showed impaired relaxant responses to SNP and to ACh (Figure 5A–D). Compared with controls, femoral vessel segments isolated from chicken embryos treated with Beta but not Dex had a significant increase in the NO‐dependent, while a significant decrease in the NO‐independent, relaxant response to ACh (Figure 5E,F). Compared with controls, femoral vessel segments isolated from chicken embryos treated with Beta but not Dex had an impaired contractile response to K^+^ and to PE (Figure 5G–J). Conversely, compared with controls, chicken embryos treated with Dex but not Beta showed an enhanced constrictor response to ET‐1 (Figure 5K,L). Similarly, there are a couple of instances (e.g., Panel 5J for) in which there may be a difference between control chicken embryos and those treated with Beta, but due to the effect size being small, we were not able to detect a significant difference in this study.

### 3.7 Lung maturation

**FIGURE 6:**
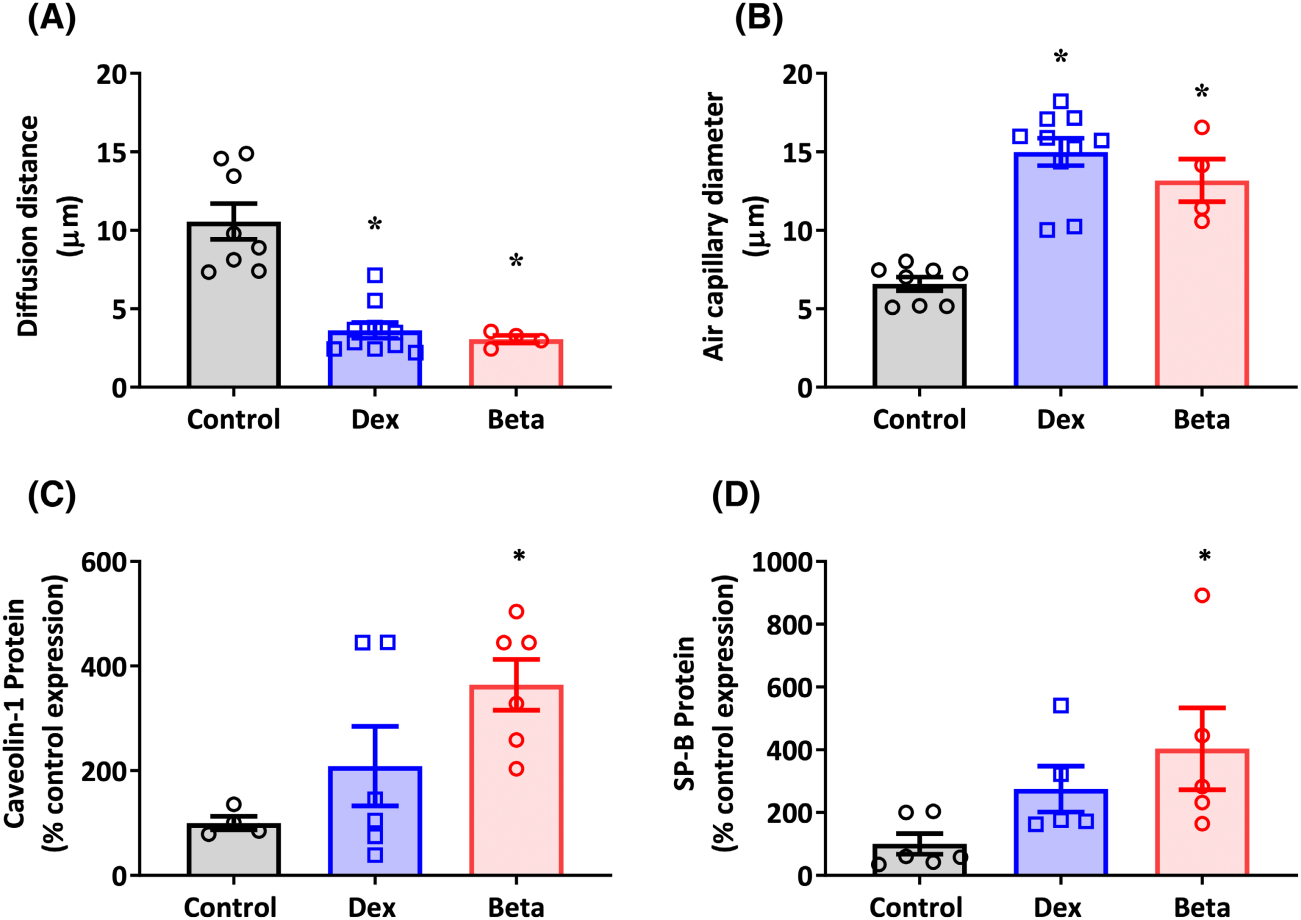
Effect of Dex and Beta on indices of lung maturation in the chicken embryo. Data are mean ± SEM for chicken embryos at day 19 of incubation following vehicle (Control: *n* = 5–8), dexamethasone (Dex: *n* = 6), or betamethasone (Beta: *n* = 6) treatment at day 14 of incubation. **(**A) Diffusion distance from air capillary lumen to nearest vessel lumen in micrometers; (B) Air capillary diameter in micrometers; (C) Protein expression of Caveolin‐1; (D) Protein expression of SP‐B. Significant differences are (*p* < .05): *versus Control; One‐way ANOVA with Tukey post hoc test.

Compared with controls, lungs from chicken embryos treated with either Dex or Beta showed a significant reduction in the diffusion distance between the air capillary to the nearest blood vessel, and a significant increase in the air capillary diameter (Figure 6A,B). Compared with controls, lungs from chicken embryos treated with Beta but not Dex showed a significant increase in the protein expression of caveolin‐1 and SP‐B (Figure 6C,D).

## 4 DISCUSSION

**FIGURE 7:**
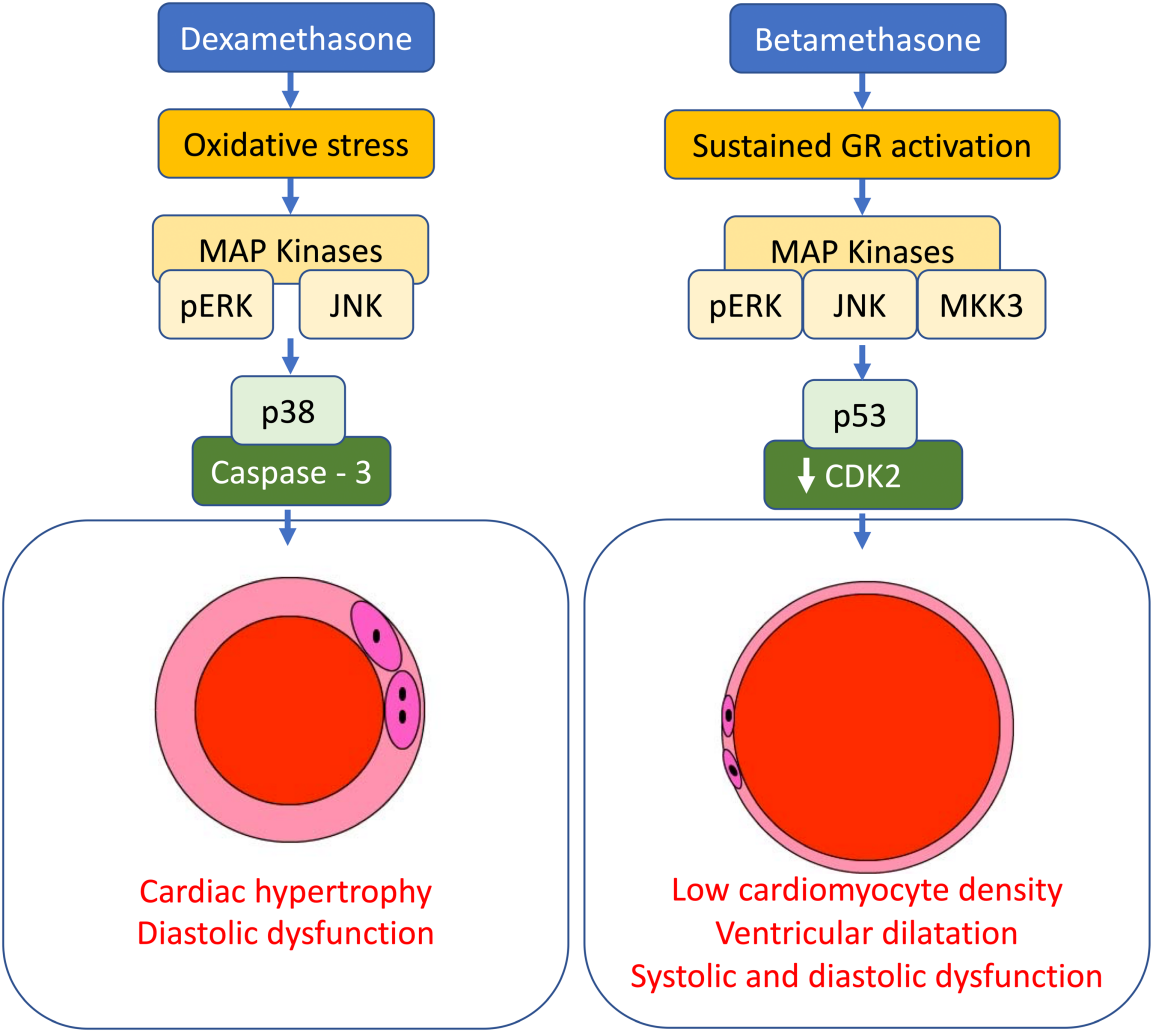
Data suggest that in Dex‐treated chicken embryos, oxidative stress signaling through pERK and JNK pathways may lead to p38‐mediated activation of caspase‐3, promoting cardiomyocyte hypertrophy. Conversely, in Beta‐treated chicken embryos, sustained activation of GR signaling through pERK/JNK/MKK3 pathways may lead to p53‐mediated repression of CDK2, triggering cellular senescence. This may explain the markedly reduced cardiomyocyte density, the dilated cardiomyopathy phenotype, yielding weak hearts with significant evidence of systolic and diastolic dysfunction in Beta‐ compared to Dex‐treated embryos.

These data show that treatment of the chicken embryo with Dex compared with Beta, at a stage of development equivalent to the 27‐week‐old human fetus, promotes differential adverse effects on embryonic growth and cardiovascular development at day 19 of incubation. Both Dex and Beta resulted in asymmetric growth restriction, with Beta having a more severe effect. At the level of the heart, both treatments promoted cellular stress and changes to the cell cycle. While Dex treatment induced indices associated with oxidative stress and GR downregulation, Beta treatment led to sustained GR activation, and it did not induce oxidative stress (Figure 7). While Beta induced a marked reduction in cardiomyocyte nuclear density, Dex resulted in cardiomyocyte hypertrophy. Only Beta resulted in significant systolic as well as diastolic dysfunction, in biventricular dilatation, and in a decrease in aortic wall thickness. In the peripheral vasculature, Beta impaired vasodilator function, while Dex resulted in greater vasoconstrictor function. Both glucocorticoids resulted in histological indices of lung maturation, however, only Beta significantly increased the lung protein expression of caveloin‐1 and SP‐B by day 19. Combined, therefore, the data support the hypothesis tested and provide evidence for differential effects of Dex compared with Beta on embryonic growth and cardiovascular development.

In precocial mammals, such as sheep, the increase in endogenous fetal plasma cortisol toward term switches tissue accretion to differentiation in many organ systems including the lung; it is therefore an established maturational signal in preparation for postnatal life.^4^, ^5^, ^45^, ^46^ In the chicken embryo, cardiomyocytes are mononucleated at term with binucleation starting after hatching.^47^ Therefore, in this study, there was evidence of an accelerated switch from accretion to differentiation in the hearts of Dex‐treated chicken embryos, showing a greater presence of binucleated cardiomyocytes at term. The hypertrophic effects of Dex on cardiomyocytes without a significant reduction in cell number may have compensated in part for the left ventricular dilatation, thereby better maintaining systolic and diastolic function in Dex‐ compared with Beta‐treated embryos. Conversely, the pronounced reduction in cardiomyocyte number without compensation in volume in Beta‐treated embryos, was associated with marked biventricular dilatation, yielding a weaker thin‐walled heart with evidence of significant systolic and diastolic dysfunction. While both synthetic glucocorticoids lead to an increase in LVEDP and the time constant of ventricular relaxation Tau (established indices of cardiac diastolic dysfunction), only Beta significantly reduced LVDP and the contractility index, measures of systolic dysfunction.^48^ This is consistent with an effect of Beta but not Dex in shortening the duration of the systolic component of the cardiac cycle. Since a fall in cardiomyocyte endowment, and evidence of systolic and diastolic dysfunction are all associated with cardiovascular mortality,^49^, ^50^ these effects of Beta compared to Dex are of potential clinical relevance for the future cardiovascular health of children exposed to antenatal glucocorticoid therapy. Any human translational effects of synthetic glucocorticoids on cardiovascular development will be highly dependent not only on the dosing regimen used but also on the circulating concentration achieved. In this study, measured circulating Dex and Beta concentrations achieved in the chicken embryo plasma were a quarter to one fifth of circulating values measured in human infants, 24 h post‐treatment.^42^, ^51^ Therefore, the effects of Dex and Beta observed on the cardiovascular system in the chicken embryo occurred at lower circulating levels achieved in preterm human babies.

Additional data in this study support a link between cellular stress, and proliferation pathways and the differential effects of Dex compared with Beta on cardiac structure and function at embryonic day 19. In Dex‐treated embryos only, there was an increase in the cardiac protein expression of HSP70 and HSP27, which are established heat shock proteins which may associate with increased oxidative stress as well as other triggers.^52^, ^53^ Responses to external stresses activate mitogen‐activated (MAP) kinase systems, including ERK and the two stress‐activated protein kinases p38 and c‐Jun N‐terminal kinase (JNK).^53^, ^54^ Endoplasmic reticulum stress has also been shown to activate chaperone proteins HSP60 and the ER protein disulfide isomerase (PDI).^53^, ^55^ ER stress may be secondary to ROS generation within the cell, therefore, in Dex‐treated embryos, oxidative stress induction of stress pathways pERK and JNK may result in p38‐mediated activation of caspase‐3, promoting cardiomyocyte hypertrophy.^53^ The elevation of HSP27 may further act to inhibit apoptosis, with instead cells halting proliferation. Conversely, in Beta‐treated embryos, there was no evidence of oxidative stress, however, similar stress indices were still present, namely pERK, pSAPK/JNK, HSP60, and PDI. That these pathways were still active despite no evidence of oxidative stress may suggest a more direct effect of GR activation on cellular stress pathways. The hearts of Beta‐treated embryos at day 19 did express a higher level of GR protein than Dex‐treated embryos. This may suggest GR downregulation in Dex‐treated embryos that is lost in the Beta treatment group. This may be a direct effect on cellular GR pools, or negative feedback on the glucocorticoid axis. This loss of GR downregulation may lead to excessive stimulation of the pathway in these hearts. This is interesting because an interplay between sustained GR stimulation and activation of kinase signaling pathways, such as pERK, JNK, and MKK3 has also been reported.^56^ In this study, hearts from Beta‐treated embryos further showed the induction of proteins that slow down cell division, such as p16 and p53^57^ and a reduced expression of the cyclin‐dependent kinase 2 (CDK2). CDK2 plays a role in G1/S transition, initiation of DNA synthesis, and exit regulation from the S phase of the cell cycle.^58^ Further, CDK2 transcriptional repression is an essential effector in p53‐mediated cellular senescence.^57^ Combined, our findings suggest that in Beta‐treated embryos, sustained activation of GR signaling through pERK/JNK/MKK3 pathways may lead to p53‐mediated repression of CDK2, triggering cellular senescence. This may explain the markedly reduced cardiomyocyte density, the dilated cardiomyopathy phenotype, yielding weak hearts with systolic and diastolic dysfunction in Beta‐treated embryos.

In the peripheral vasculature, dilator reactivity is a result of the integration of signals delivered to the vascular smooth muscle cells (vSMC) and/or the endothelium. Acetylcholine is known to induce vasodilation via muscarinic receptors activating a variety of pathways including NO production by eNOS, prostacyclin production by cyclo‐oxygenase‐1 (COX‐1), and the induction of other vasomotor agents such as H_2_S.^59^ NO rapidly diffuses into the vSMCs inducing further vasodilatation by enhancing Ca^2+^ reuptake, reducing cytosolic Ca^2+^ levels, and opening hyperpolarizing K^+^ channels.^60^, ^61^, ^62^ In this study, the reduction of both SNP and Ach‐mediated vasodilation in the Beta‐treated, but not Dex‐treated embryos suggests a serious impairment in vascular reactivity. Dissecting the Ach‐induced vasodilator mechanisms demonstrated that the dilation mediated by NO was actually increased in the Beta embryos, despite the overall dilation still being impaired relative to Dex and control embryos. The NO‐independent mechanisms of dilation are also impaired in these animals, suggesting an endothelial as well as a vSMC dysfunction. Similarly, Anwar et al.^63^ reported reduced dilator reactivity in femoral vessels isolated from fetal sheep treated with Beta. Additional experiments in this study investigating vasoconstrictor function also showed divergent effects in Dex‐, compared with Beta‐treated, chicken embryos. Enhanced vasoconstrictor reactivity to ET has also been reported in femoral vessels isolated from fetal sheep treated with Dex.^64^, ^65^ Heightened responsiveness to depolarization by K+ may suggest hypertrophy or increased proliferation of vSMCs following Dex treatment which is lacking with Beta treatment.

In this study, we demonstrate that both Dex and Beta have a significant effect on maturation of the embryonic lungs. Both glucocorticoids significantly altered histological indices of maturation, such as decreased air diffusion distance and increased air capillary diameter. However, only Beta treatment significantly upregulated surfactant proteins SP‐B and Caveolin‐1 by embryonic day 19. Previous work assessing the development of chicken lungs has demonstrated a late developmental switch toward production of type‐II pneumocytes, increased production of surfactant lipids and proteins (including SP‐B and caveolin‐1), and histological changes, which can be induced in vitro via glucocorticoid exposure.^66^, ^67^, ^68^, ^69^ This study supports that exposure to exogenous glucocorticoids stimulated this developmental switch in the chicken embryo earlier in Beta‐treated embryos. Other studies assessing the efficacy of Dex compared with Beta in terms of lung maturation have previously suggested that Beta may be more potent in reducing risk of pulmonary complications in preterm infants, which may be due to accelerating lung maturation at a greater rate than Dex.^70^ Studies of oral AGT administration have also demonstrated that Dex has a less significant effect on lung compliance than the same dose of Beta.^17^, ^18^ Additionally, in the sheep, maternal intramuscular injection with Beta resulted in increased mRNA of surfactant proteins, whereas injection with Dex did not.^16^, ^71^

Distinct effects on the developing cardiovascular system may seem at odds with two drugs that are stereoisomers and should act very similarly in the body. In vitro, it has been shown that Dex can activate noncanonical GR signaling with a greater potency than Beta; therefore, noncanonical stimulation may explain some of the divergent effects between the two steroids.^56^ The other likely difference is the pharmacokinetics of the two drugs, which are produced as differing prodrug formulations. Dexamethasone typically is available as a sodium phosphate formulation, and betamethasone as a 50:50 ratio of sodium phosphate and acetate prodrug formulations, the most common brand being Celestone Soluspan.^7^ It is known that the sodium phosphate formulation is more rapidly activated by endogenous phosphatases within the body. Evidence from maternal glucocorticoid administration in sheep has demonstrated that Dexa or Beta phosphate administration, in human clinically relevant doses and dose regimens, results in a rapid peak of active glucocorticoid in the fetal circulation at 1–2 h, and rapidly decreases to basal level by 24 h.^17^, ^72^ Conversely, maternal administration with Beta acetate does not produce a rapid large peak of active glucocorticoid, but a sustained smaller increase that is maximal at 8.5 h postinjection, and is still detectable after 24 h.^17^

## 5 PERSPECTIVES

In this study, we show that Dex and Beta have differential detrimental off‐target effects on the developing cardiovascular system in the late incubation chicken embryo, independent of effects on the mother and/or placenta. These results contribute important knowledge to consider in relation to balancing beneficial short‐term effects of antenatal glucocorticoid therapy versus potential long‐term adverse cardiovascular effects. They provide a platform from which further experiments in mammalian models can be designed with the aim of identifying antenatal glucocorticoid therapy regimes that maintain beneficial effects on the lung while minimizing adverse off‐target effects on the developing cardiovascular system, thereby making it safer for the treatment of preterm birth.^5^, ^29^

## AUTHOR CONTRIBUTIONS

Dino Giussani, Tessa Garrud, Susan Ozanne, and Jan Derks designed the research. Tessa Garrud, Noor Teulings, Youguo Niu, Katie Skeffington, Christian Beck, Nozomi Itani, Fiona Conlon, Kimberley Botting, Lisa Nicholas, and Wen Tong performed the research and analyzed the data. Tessa Garrud and Dino Giussani drafted the article. All authors approved the article.

## FUNDING INFORMATION

The work was supported by the British Heart Foundation (DAG: PG/10/99/28656); The Lister Institute (DAG: RG85891); and the Department of Perinatal Medicine, University Medical Centre, Utrecht, Netherlands (JBD and NT). SEO is a member of the MRC Metabolic Diseases Unit (MC_UU_00014/4).

## DISCLOSURES

There are no conflicts of interest or disclosures.

## Supporting information


Table S1.

Table S2.

## Data Availability

The data that support the findings of this study are within the article and/or supplementary material of this article.
