# Supplementary material for: Molecular mechanisms underlying adverse effects of dexamethasone and betamethasone in the developing cardiovascular system
Source: FASEB J. 2023 May 3;37(6):e22887. doi: 10.1096/fj.202200676RR (PMC10946807; doi:10.1096/fj.202200676RR)
Supplement: Supplementary file 1 — Table S1. [file FSB2-37-0-s001.docx]

**Major Resources Table**

In order to allow validation and replication of experiments, all essential research materials listed in the Methods should be included in the Major Resources Table below. Authors are encouraged to use public repositories for protocols, data, code, and other materials and provide persistent identifiers and/or links to repositories when available. Authors may add or delete rows as needed.

**Animals (in vivo studies)**

| **Species** | **Vendor or Source** | **Background Strain** | **Sex** | **Persistent ID / URL** |
| --- | --- | --- | --- | --- |
| Gallus Gallus (chicken) | Medeggs, Henry Stewart & co. (United Kingdom) | Bovans Brown | Male and female | https://www.medeggs.com/products |
|  |  |  |  |  |
|  |  |  |  |  |

**Genetically Modified Animals**

|  | **Species** | **Vendor or Source** | **Background Strain** | **Other Information** | **Persistent ID / URL** |
| --- | --- | --- | --- | --- | --- |
| **Parent - Male** |  |  |  |  |  |
| **Parent - Female** |  |  |  |  |  |

**Antibodies**

| **Target antigen** | **Vendor or Source** | **Catalog #** | **Working concentration** | **Lot # (preferred but not required)** | **Persistent ID / URL** |
| --- | --- | --- | --- | --- | --- |
| HSP27 | Cell Signaling Technology | 2402S | 1:1000 |  | https://www.cellsignal.com/products/primary-antibodies/hsp27-g31-mouse-mab/2402?site-search-type=Products&N=4294956287&Ntt=2402s&fromPage=plp&_requestid=183660 |
| HSP60 | Abcam | Ab46798 | 1:500 |  | https://www.abcam.com/hsp60-antibody-ab46798.html |
| HSP70 | Cell Signaling Technology | 4872 | 1:500 |  | https://www.cellsignal.com/products/primary-antibodies/hsp70-antibody/4872 |
| Phosphorylated ERK1/2 | Cell Signaling Technology | 9101S | 1:250 |  | https://www.cellsignal.com/products/primary-antibodies/phospho-p44-42-mapk-erk1-2-thr202-tyr204-antibody/9101?site-search-type=Products&N=4294956287&Ntt=9101s&fromPage=plp&_requestid=183753 |
| ERK1/2 | Cell Signaling Technology | 9102 | 1:500 |  | https://www.cellsignal.com/products/primary-antibodies/p44-42-mapk-erk1-2-antibody/9102 |
| SAPK/JNK | Cell Signaling Technology | 9252S | 1:1000 |  | https://www.cellsignal.com/products/primary-antibodies/sapk-jnk-antibody/9252?site-search-type=Products&N=4294956287&Ntt=sapk+jnk&fromPage=plp |
| Phosphorylated SAPK/JNK | Cell Signaling Technology | 9251S | 1:1000 |  | https://www.cellsignal.com/products/primary-antibodies/phospho-sapk-jnk-thr183-tyr185-antibody/9251?_=1646265022877&Ntt=sapk%20jnk&tahead=true |
| PDI | Abcam | Ab3672 | 1:1000 |  | https://www.abcam.com/bin1-antibody-ab27796.html |
| GR | Santa Cruz Biotechnology | Sc-393232 | 1:500 |  | https://www.scbt.com/p/gr-antibody-g-5?requestFrom=search |
| Cleaved caspase-3 | BD Biosciences | 564094 | 1:500 |  | https://www.bdbiosciences.com/en-us/products/reagents/flow-cytometry-reagents/research-reagents/single-color-antibodies-ruo/bv605-rabbit-anti-active-caspase-3.564094 |
| SP-B | Stratech | 365148 | 1:500 |  | https://www.stratech.co.uk/products/365148-USB/ |
| Caveolin-1 | Thermo Fisher | PA5-17447 | 1:1000 |  | https://www.thermofisher.com/antibody/product/Caveolin-1-Antibody-Polyclonal/PA5-17447 |

**DNA/cDNA Clones**

| **Clone Name** | **Sequence** | **Source / Repository** | **Persistent ID / URL** |
| --- | --- | --- | --- |
|  |  |  |  |
|  |  |  |  |
|  |  |  |  |

**Cultured Cells**

| **Name** | **Vendor or Source** | **Sex (F, M, or unknown)** | **Persistent ID / URL** |
| --- | --- | --- | --- |
|  |  |  |  |
|  |  |  |  |
|  |  |  |  |

**Data & Code Availability**

| **Description** | **Source / Repository** | **Persistent ID / URL** |
| --- | --- | --- |
|  |  |  |
|  |  |  |
|  |  |  |

**Other**

| **Description** | **Source / Repository** | **Persistent ID / URL** |
| --- | --- | --- |
|  |  |  |
|  |  |  |
|  |  |  |
