# Supplementary material for: Molecular mechanisms underlying adverse effects of dexamethasone and betamethasone in the developing cardiovascular system
Source: FASEB J. 2023 May 3;37(6):e22887. doi: 10.1096/fj.202200676RR (PMC10946807; doi:10.1096/fj.202200676RR)
Supplement: Supplementary file 2 — Table S2. [file FSB2-37-0-s002.pdf]

Molecular mechanisms underlying adverse effects of Dexamethasone and Betamethasone in the developing cardiovascular system

Tessa A.C. Garrud PhD[1], Noor E.W.D. Teulings MD PhD[2], Youguo Niu MD PhD[1], Katie L. Skeffington PhD[1], Christian Beck PhD[1], Nozomi Itani PhD[1], Fiona Conlon BSc[1], Kimberley J. Botting PhD[1], Lisa M. Nicholas PhD[2], Wen Tong PhD[1], Jan B. Derks MD PhD[3], Susan E. Ozanne PhD[2,4,5,6] & Dino A. Giussani PhD[1,4,5,6]

Affiliations:

[1]Department of Physiology, Development and Neuroscience, University of Cambridge, Cambridge, UK.

[2]Institute of Metabolic Science-Metabolic Research Laboratories & MRC Metabolic Diseases Unit, University of Cambridge, Addenbrooke's Hospital, Cambridge, UK.

[3]Department of Perinatal Medicine, University Medical Centre, Utrecht, Netherlands.

[4]BHF Cardiovascular Centre for Research Excellence, University of Cambridge, Cambridge, UK.

[5]Strategic Research Initiative in Reproduction, University of Cambridge, Cambridge, UK.

[6]Centre for Trophoblast Research, University of Cambridge, Cambridge, UK.

Correspondence: Professor Dino A. Giussani, PhD
Department of Physiology, Development and Neuroscience
University of Cambridge, Cambridge, CB2 3EG, UK
Tel: +44 1223 333894
Fax: +44 1223 333840
E-mail: dag26@cam.ac.uk

Category: Article

Supplementary Table 1. Forward and reverse primer sequences

Gene	Forward Primer	Reverse Primer
MKK3	CTACTTGGTGGACTCGGTGG	CGACTTCACGTTGTAGCCCT
p16	GAAGCGCGGAAGAAGACACC	GGCAACCGACGGAATGTTTG
p38	CTACTCCCCCTGCCACTTTTT	GTACACACCAGCCACCTACA
p53	GTGGGCTCTGACTGTACCAC	GCCCTCCAGTGTAAGGATG
cdk2	TCTTCCGTATCTTCCGCACG	ATGCGCTTGTTGGGATCGTA
